# Neuropsychology of Consciousness: Some History and a Few New Trends

**DOI:** 10.3389/fpsyg.2019.00050

**Published:** 2019-01-30

**Authors:** Giovanni Berlucchi, Carlo Alberto Marzi

**Affiliations:** Department of Neurosciences, Biomedicine and Movement, University of Verona, Verona, Italy

**Keywords:** neuropsychology, consciousness and unconsciousness, arousal and content, hippocampus, thinking – dreaming

## Abstract

Consciousness is a global activity of the nervous system. Its physiological and pathological mechanisms have been studied in relation to the natural sleep-wake cycle and various forms of normal or morbid unconsciousness, mainly in neurophysiology and clinical neurology. Neuropsychology has been more interested in specific higher brain functions, such as perception and memory and their disorders, rather than in consciousness *per se*. However, neuropsychology has been at the forefront in the identification of conscious and unconscious components in the processing of sensory and mnestic information. The present review describes some historical steps in the formulation of consciousness as a global brain function with arousal and content as principal ingredients, respectively, instantiated in the subcortex and the neocortex. It then reports a few fresh developments in neuropsychology and cognitive neuroscience which emphasize the importance of the hippocampus for thinking and dreaming. Non-neocortical structures may contribute to the contents of consciousness more than previously believed.

## Introduction

In neuropsychology, localization of psychological functions in the brain has been classically based on the observation that patients carrying a lesion in a particular cerebral region exhibit a loss or disorders of a particular psychological ability, while other abilities are preserved. Speech has been localized in the frontal lobe of the left hemisphere because lesions in that region cause expressive aphasia, whereas similar destructions in the right hemisphere have no such effect. Similarly, certain visual perceptual abilities can be localized in the occipito-temporal cortex because they are disturbed by lesions in that part of the cortex but not by lesions in other cortical regions. Of course, such localizations do not imply that any given psychological function can unfold only in a given part of the brain: they only mean that a specific part of the brain houses a “hub,” a crucial focus of activity, in the overall cerebral organization of that function. Attempts at localizing a hub for consciousness in the brain on the basis of the effects of brain lesions or dysfunctions that lead to unconsciousness are probably misconceived, insofar as consciousness is best seen as a global function of the brain in action which can be interfered with by nervous tissue damage or malfunctioning from a variety of factors. Large portions of the brain which are certainly known to be involved in consciousness can be removed without causing loss of consciousness, as in the case of the ablation of a whole cerebral hemisphere. If there are in the brain “master switches” which can turn consciousness on and off (Blumenfeld, 2014), these must be able to change the entire cerebral organization at once.

## Formulation of Consciousness

The philosopher Searle (1993) defines consciousness as “those subjective states of sentience or awareness that begin when one awakes in the morning from a dreamless sleep and continue throughout the day until one goes to sleep at night or falls into a coma, or dies, or otherwise becomes, as one would say, ‘unconscious.”’ While this terse definition captures many essential aspects of the natural dichotomy between consciousness and unconsciousness, as well as their relations with the physiological sleep-wake cycle and with the pathology of consciousness, it requires several qualifications based on current neuroscientific knowledge. It is true that there is a strong association between wakefulness and consciousness, but to be awake does not necessarily mean to be conscious, and to be asleep does not necessarily mean to be unconscious. Brain damaged patients in the vegetative state are persistently unaware of themselves and their environment, despite exhibiting irregular sleep-wake cycles whereby waking occurs with eye opening, but without any meaningful contact with the environment. Brief dissociations between consciousness and a wakeful appearance characterize the absence seizures or the complex partial seizures of epileptic patients and can be interpreted as momentary vegetative states (Plum and Schiff, 2003), although the presence of a minimal form of consciousness in at least some cases cannot be excluded (Bayne, 2011).

In everyday life, wakeful healthy individuals appear continuously conscious to themselves and to others (although of course many of their purposeful actions are carried out without the intervention of consciousness), but there is evidence for the occurrence of occasional “mind-blanking” moments of behavioral inaction and inability to report subjective inner experiences (Ward and Wegner, 2013). In turn, sleep can hardly be equated with unconsciousness, given that reportable dreams occupy parts of all stages of sleep, and not only of the REM (rapid eye movement) stage which in the past had been specifically linked to dreaming. By current estimates, dreaming takes up 80% of total REM sleep time and 50% of total non-REM sleep time, which means that on average one can be considered unconscious during only 44% of the time of a night’s sleep (Cipolli et al., 2017; Siclari et al., 2017). Paradoxically, dreaming consciousness is probably absent in somnambulism, such that perpetrators of crimes during sleepwalking have been absolved on account of their presumed temporary unconsciousness (Kannape et al., 2017).

In normal everyday life, consciousness and unconsciousness are two distinct states of the whole organism, depending on different active modes of brain functioning, which alternate in some relation with the sleep-wake cycle but are partially independent of it. The normal brain is always active, and the natural unconsciousness of dreamless sleep is a physiological mode of brain functioning, as contrasted with the pathological modes of brain dysfunction underlying the unconsciousness of coma. The main behavioral difference between the physiological unconsciousness of dreamless sleep and the pathological unconsciousness of coma is that a healthy sleeping individual can always be aroused and brought back to conscious wakefulness by sensory stimuli of appropriate intensity, whereas a comatose patient cannot. Pharmacological unconsciousness induced by general anesthesia mimics coma, except for the quick return of arousability with the wearing off of the effects of the anesthetic agent (Brown et al., 2010).

## Some Historical Landmarks in the Neurology of Arousal, Wakefulness and Consciousness

The neurophysiological mechanisms of arousal were discovered by Moruzzi and Magoun (1949) by inducing behavioral and electroencephalographic arousal reactions in lightly anesthetized cats upon electrical stimulation of the bulbo-ponto-mesencephalic reticular formation. They attributed the natural arousal reaction from sensory stimuli to the activation of the reticular formation and its prolongation in the hypothalamus and thalamus, resulting in the activation of the whole cerebral cortex. They also suggested that a continuous reticular activity, whether of endogenous or exogenous origin, could be a major factor in the maintenance of the waking state. By damaging the ascending projections of the reticular formation, Magoun and collaborators rendered cats and macaques comatose, confirming that a waking brain is the result of a continuous reticular activating action on the cerebral cortex (Magoun, 1952). The neurosurgeon French, a collaborator of Magoun, extended the results to human wakefulness and consciousness by studying a few patients with prolonged loss of consciousness after lesions of either the cephalic end of the brainstem reticular formation, or of its subcortical radiation, or of the entire cerebral cortex by meningoencephalitis (French, 1952). He was the first to call attention to “a possible conflict in terminology denoting the physiological and pathological conscious conditions of sleep and coma,” because he had observed in his patients occasional brief periods of wakefulness with open eyes which were devoid of any evidence of conscious awareness. He thus implied that wakefulness is not necessarily a proof of consciousness, and advised against considering the reticular activating system as a center of wakefulness or consciousness, insofar as the manifestations of its activity are expressed only through its influences on other subcortical structures, such as the posterior hypothalamus, which had long been implicated in disorders of consciousness, or on the entire cortex (French, 1952).

In the 1960s and 1970s century some neurophysiological mechanisms of sleep and waking were identified in experimental animals (Moruzzi, 1963, 1972) and their results were used in the interpretation of major disturbances of consciousness in humans. Two syndromes characterized by clear dissociations between behavior and consciousness were described and named in brain damaged patients. Jennett and Plum (1972) gave the name “persistent vegetative state” (now also called unresponsive wakefulness) to a syndrome whose essential component “is the absence of any adaptive response to the external environment, the absence of any evidence of a functioning mind which is either receiving or projecting information, in a patient who has long periods of wakefulness.” These waking periods, attested by the opening of the eyes, whether spontaneous or elicited by sensory stimulation, differentiate the vegetative state from coma, in which the eyes remain permanently closed even under strong stimulations. In vegetative state patients, diencephalic and brainstem arousal mechanisms appear sufficiently functional for supporting a behavioral expression of wakefulness, but conscious contents are lacking because of widespread cortical damage or due to a disconnection between the subcortical arousal mechanisms and the cerebral cortex. In Jennett and Plum’s (1972) words, “common to all patients in this vegetative, mindless state is that, as best can be judged behaviorally, the cerebral cortex is not functioning, whether the lesion be in the cerebral cortex itself, in subcortical structures, the brain-stem, or in all these sites.” In a localizing attempt, Plum and Posner (1980) famously argued that consciousness has two components, content and arousal, the first mediated by unique combinations of local cortical circuits specialized for different stimuli, the second depending on brainstem and diencephalic pathways that regulate the overall level of cortical function and hence the level of consciousness.

The locked-in syndrome, first described by Plum and Posner (1966), is usually caused by pontine lesions that produce an almost complete motor de-efferentation by interrupting the cortico-spinal and cortico-bulbar components of the pyramidal tract, resulting in tetraplegia and inability to speak (Herculano-Houzel et al., 2016). Voluntary palpebral and vertical eye movements may be preserved and may be used for a coded communication based on blinking or up and down ocular movements, revealing the existence of a fully preserved conscious awareness and near-normal sensory and cognitive functions. An animal model of the human locked-in syndrome (Ikegami et al., 1977; Zernicki et al., 1978; Berlucchi, 2017) is the midpontine pretrigeminal cat (Moruzzi, 1963, 1972), in which a disconnection from lower brain stem hypnogenic neurons (Berlucchi et al., 1964; Anaclet and Fuller, 2017) disinhibits the arousal systems.

The differential diagnosis between the vegetative state, the minimally conscious state (as defined by Giacino et al., 2002) and the locked-in syndrome is subject to a high error rate (Gill-Thwaites, 2006; Schnakers et al., 2009; Wade, 2018). For example, some locked-in patients are considered unconscious because their eyes and eyelids are also paralyzed, thus making communication impossible. In the last two decades, the possible presence of consciousness in totally paralyzed, non-communicating patients has been investigated by exploiting the capacity of a few of these patients to modulate their brain activities, as assessed with neuroimaging or electrophysiological techniques, in response to commands or to engaging cognitive stimulation (Owen, 2013; Bayne et al., 2017; Graham et al., 2018). When present, such cerebral, non-behavioral evidence for consciousness can help reclassify patients previously supposed to be in a vegetative state as minimally conscious or even as functionally locked-in. To borrow one of Jennett and Plum (1972) expressions, these patients demonstrate the possession of a functioning conscious mind by projecting information as patterns of brain activity. Very recent findings suggest that different states of consciousness can be discriminated in clinical practice on the basis of machine-analyzed signals extracted from the electroencephalogram (Engemann et al., 2018).

## Cortex Versus Subcortex

The famous neurosurgeon Penfield (1978) has written that “to suppose that consciousness or the mind has localization is a failure to understand neurophysiology” (page 109). Nevertheless, he has also postulated that a centrencephalic system, more or less coincident with the higher brain stem and hypothalamus, contains the nervous mechanisms “which are prerequisite to intellectual activity ... and the initiation of the planned action of the conscious man” (Penfield, 1954). Most of his contemporary neurologists and neurosurgeons disagreed with him by conceding to the brainstem reticular system at most a menial role, metaphorically equated to that of janitors who warm up class-rooms and laboratories in a University (Levin, 1960). Granting that in an intact brain the cortex plays a major role in consciousness, to regard the ascending reticular system merely as an agent of arousal, an “energizer” concerned solely with maintaining the general excitability of the cortex, is a fallacy (Moruzzi, 1972). Indeed, after Moruzzi (1958) had criticized the concept of a single unitary arousal system on theoretical grounds (Berlucchi, 1997), the concept was made untenable by the discovery that in addition to the “classic” reticular ascending projections, which most probably use glutamate as their synaptic transmitter, other multiple ascending projections from the brainstem, the hypothalamus and the basal forebrain use other transmitters to modulate the activities of the thalamus and the cortex (Jones, 2011; Brown et al., 2012; Saper and Fuller, 2017). These multiple systems include monoaminergic projections from the pontine locus ceruleus, cholinergic projections from the ponto-mesencephalic latero-dorsal and pedunculo-pontine nuclei and from the basal forebrain, serotoninergic projections from the mesencephalic and pontine raphe nuclei, histaminergic projections from the tuberomammillary nucleus of the posterior hypothalamus and glutamatergic projections from the supramammillary nucleus of the lateral hypothalamus. Further, there are peptidergic projections to the forebrain and to all other ascending systems from lateral and posterior hypothalamic neurons which use the orexin peptide as a neurotransmitter. In experimental animals all these systems are active during waking and silent during sleep, except for the ponto-mesencephalic cholinergic projections which become active also during REM sleep. Each of these systems alone is sufficient for sustaining wakefulness, and none of them alone is necessary for that purpose except orexin, the absence of which is a cause of narcolepsy (Jones, 2011). So many arousing system working in parallel may seem redundant, but their collective activity is orchestrated, at least partly, by orexin, so that each of them can function in a different manner in different emotional and motivational conditions, thus possibly influencing some dimension of consciousness. Lesions of the rostral brainstem and posterior diencephalon which result in coma in experimental animals and humans alike probably destroy the ascending projections of all arousal systems and interfere with homeostatic regulation (Parvizi and Damasio, 2001). However, coma has also been attributed to small lesions of the rostral laterodorsal pontine tegmentum, projecting to cortical areas and neurons thought to be critical for consciousness (Fischer et al., 2016), and sudden disruption of consciousness has been produced with electrical stimulation of the left claustrum and anterior insula (Koubeissi et al., 2014). Giacino et al. (2014) have proposed that a common mechanism in disorder of consciousness may be the downregulation of an anterior forebrain mesocircuit, including thalamocortical and thalamostriatal connections focused on the central thalamus, with a possible contribution from the pedunculopontine nucleus.

The old question of whether processes implementing conscious contents occur only in the cortex or to some extent also subcortically is at the center of the current debate between affective and cognitive neuroscience. As detailed in a recent discussion (Panksepp et al., 2017; see also Adolph and Anderson, 2018), affective neuroscience places the ancestral indicators of affective consciousness in evolutionary ancient non-cortical survival networks, and maintains that subcortical activation is both necessary and sufficient for primitive affective experience. In contrast, cognitive neuroscience views all types of consciousness as involving the same global cortical broadcasting mechanism and holds that subcortical processes are necessary but not sufficient for affective experience. Cognitive neuroscience concedes that the cerebral cortex alone, without interaction with subcortical processes, cannot sustain consciousness, but insists that absence of a cortex implies absence of consciousness. In a survey entitled “consciousness without a cerebral cortex: a challenge for neuroscience and medicine,” Merker (2007) has forcefully argued that an upper brainstem system, extending from the roof of the midbrain to the basal diencephalon, serves by itself as a medium for the elaboration of conscious contents. In his view this system accounts for the elaborate goal-directed behaviors of decorticated rodents, as well as for the presence of conscious experiences in some hydranencephalic children, born without most of the cerebral cortex because of massive loss of hemispheric tissue during gestation. Both ordinary neurological examination and the reports of primary caregivers attest that these children, though affected by severe sensory deficits such as blindness, are capable of experiencing pain, discomfort and suffering, but also other hedonic states including comfort, pleasure and joy (Aleman and Merker, 2014). The possibility that the emergence of consciousness can precede the development and maturation of the cortex has long been advocated by Trevarthen and Reddy (2017) on the basis of the presence in fetuses and premature newborns of an exploratory search for, and an appropriate reaction to, sensory stimuli, along with motor expressions of distress, curiosity, or pleasure, clearly aimed at the social communication of interests and feelings.

On the other hand, current authoritative theories of consciousness, such as the global neuronal workspace theory (Dehaene and Changeux, 2011) and the integrated information theory (Tononi et al., 2016), keep alive the concept that the terms arousal and waking refer to a global regulation of cerebral organization by brainstem and diencephalic activities, whereas conscious contents depend on local and specific cortical or thalamo-cortical organizations. Hill and Tononi (2005) have provided a large-scale computer model that accounts for sleep-wake transitions in brain activity in terms of specific changes at the neuronal level in the thalamocortical, corticothalamic, and corticocortical connections. According to the model, both waking and physiological sleep require a specific balance of excitation and inhibition in these connections, a balance which may be disrupted in severe disorders of consciousness. By measuring the electroencephalographic response to transcranial magnetic stimulation, Rosanova et al. (2018) have recently documented in awake vegetative state patients a pathological tendency of intact cortical circuits to fall into silence upon receiving an input, at variance with the complex pattern of propagation and interactions set up in the cortex of healthy awake individuals by the initial activation, but similar to the non-propagated cortical reaction observed in unconscious healthy individuals during natural sleep. As one possible cause for the presence of cortical responses typical of the normal sleeping brain in awake but unconscious brain damaged patients, the authors mention the possibility that a diffuse axonal injury deprives the cortical circuits of a critical amount of fibers of the ascending activating systems.

In this connection, Koch et al. (2016) distinguish two neuronal correlates of consciousness, a full correlate, i.e., the neural substrate supporting experience in general, irrespective of its specific content, and a content-specific correlate, i.e., the neural substrate supporting a particular content of experience – for example, faces, whether seen, dreamed or imagined. According to them the arousal systems are background factors that enable consciousness by ensuring an adequate excitability of the neuronal correlates of consciousness, but do not contribute directly to the content of experience. As for the possible nature of the neuronal cortical correlates of conscious contents, recordings in epileptic patients have demonstrated the existence in the human medial temporal cortex of single neurons representing specific objects or events or persons (Quian Quiroga et al., 2013; Quian Quiroga, 2016), corresponding to the gnostic units of Konorski (1967) or to the cardinal cells of Barlow (1972). However, many believe that aggregations of neurons like the cell assemblies proposed by Hebb (1949) are more likely to constitute the correlates of conscious contents (e.g., Huyck and Passmore, 2013; Eichenbaum, 2018), although the possible contribution of highly specialized single neurons, like the so-called grandmother neurons (Gross, 2002), is not ruled out completely (Bowers, 2009).

Traditionally, neuropsychology has been more interested in the brain lesions that cause fractional losses of consciousness, such as various forms of agnosias, rather than in the brain dysfunction which entail a total loss of consciousness. Interest for the study of consciousness in neuropsychology is typically attested by the many dissociations discovered by neuropsychologists whereby residual cognitive abilities following brain damage occur in the absence of acknowledged awareness by the patients, as exemplified by blindsight, implicit memories in amnesia, hidden information processing in unilateral neglect, covert recognition of faces in prosopagnosia, and so forth. In blindsight, for example, some patients who are blind in one half of the visual field as a result of a cortical lesion can detect or discriminate visual stimuli of which they are utterly unaware (Weiskrantz, 1998; Marzi, 1999), and the “Gestalt” configurations of visual stimuli can be implicitly detected even when such stimuli are presented to a completely decorticated cerebral hemisphere (Georgy et al., 2016). Other dissociations allowing an at least partial identification of the neural bases of conscious and unconscious aspects of vision have been examined in neuropsychological experiments on healthy participants, using for example binocular rivalry or “masking” paradigms allowing a comparison between supraliminal and subliminal stimuli in vision or other perceptual modalities (Seth, 2018). In the following we will deal with a new neuropsychological approach to the study of the neural bases of consciousness, focusing on the hippocampus, thinking and dreaming.

## Hippocampus in Thinking and Dreaming

Intuitively most contents of consciousness correspond to the perceived objects and events of the present environment, but there also exist internally generated contents that are not directly driven by immediate perceptual input. These contents of consciousness, or thoughts, can reflect the present situation as well as dissociate themselves from the “here and now” by referring to the remembered past, or to the foreseen future, or to entirely fictitious scenarios. Thoughts are produced during mental activities, variously named task-dependent and task-independent thinking, daydreaming, mind wandering, and mental time travel, which have been calculated to occupy as much as 30–50% of our waking mental activities, ranging from future planning, problem-solving and creativity to rumination and metacognition. Mental time travel involves a metaphorical navigation of the past as well of the future, and considerable evidence points to the hippocampus as a crucial brain structure not only for the actual navigation of the current environment, but also for the mental navigation of time past and future (Corballis, 2015; Smallwood and Schooler, 2015; Christoff et al., 2016; Fox and Christoff, 2018). The manners in which the hippocampus contributes to memory, visual imagery, navigation and cognition have been spelled out recently by Lisman et al. (2017). The Nobel prize winning discovery of place cells in the hippocampus and grid cells in the entorhinal cortex of rats (Moser et al., 2015; Hartley et al., 2017) has revealed the existence of a neural system that allows the navigation not only of the present environment, but also of the record of an animal’s life (Cohen, 2015). Activities of neurons and neuronal assemblies in the hippocampal regions can tell the story of where the animal has been, where it will or might go, and which stimuli have been encountered in various locations. As elaborated by Nadel and Ranganath (in Lisman et al., 2017), the hippocampus has presumably evolved as a brain mechanism that organizes experiences according to their spatial and temporal relationships, not only by specifying the locations of foraging sites and potential predators, but also by enabling enduring and meaningful representations of these locations in a spatio-temporal context. In their words, “the hippocampal map can support memory for the location of a tree that only has fruits in the summertime, or the site of a water source that is frequented by predators at night but safe during the daytime.”

In humans, brain imaging shows that thinking about the past and future episodes activates a common network in the brain of which the hippocampus is a major component (Addis et al., 2007; Beaty et al., 2018). Medial temporal lobe lesions including the hippocampus cause a most severe anterograde amnesia combined with a variable retrograde amnesia, as well as an inability to plan future actions. Many years ago, in their first description of a human Klüver-Bucy syndrome due to a two-stage extensive bilateral removal of the temporal lobes of an epileptic patient, Terzian and Dalle Ore (1955) prophetically wrote that the operation, though successful in improving the epileptic condition, had left the patient without a past to remember and consequently with no future to look forward to. The purest case of amnesia caused by a bilateral medial temporal surgical ablation is the late Henry Molaison, originally studied by neuropsychologist Brenda Milner (Milner and Klein, 2016). Milner’s former student Suzanne Corkin has published a best-selling biography of this patient, the title of which, “Permanent present tense,” refers to the fact that after his brain operation the patient’s consciousness was totally stuck to the “here and now” (Corkin, 2013). The incapacity for mental time travel of amnesic patients with hippocampal lesions cannot be attributed to dementia because of their spared sense of the self, as manifest in the appropriate use of personal pronouns and adjectives in verbal communication. Nor do hippocampal lesions destroy the objective cognition of physical time as measured by clocks and calendars, since patients with these lesions can conceive about either past and future, although for them both past and future are empty of actual and foreseeable personal episodes. In brief, amnesic patients with hippocampal lesions appear to possess a factual, semantic knowledge of a physical time, whereby present is preceded by past and followed by future, but are unable to travel in it with their mind because they cannot retrieve personal episodes from their past or imagine themselves in future episodes. In a recent experiment (McCormick et al., 2018), spontaneous thinking was studied in patients affected by small hippocampal lesions from limbic encephalitis and deficits of episodic memory, though less severe than those of Henry Molaison. Spontaneous thoughts of these patients were compared with those of healthy controls by systematic sampling in both groups and by asking participants about the thought content and whether the thought concerned the present moment or past or future time points at different distances from the present. Though perfectly able to entertain spontaneous thoughts detached from the current external environment, patients with hippocampal damage reported conscious contents markedly different from those of healthy controls. While the controls^TM^ thoughts could concern past, present, and future, and were couched primarily in terms of detailed visual episodes, the patients’ thoughts were anchored in the present, verbally mediated and devoid of visual images. In the authors’ words, these findings “expose the hippocampus as a key pillar in the neural architecture of mind-wandering and reveal its impact beyond episodic memory, placing it at the heart of our mental life” (McCormick et al., 2018).

There are various phenomenological and conceptual parallels between spontaneous thoughts, particularly during mind wandering, on one hand, and the contents of dreams during sleep on the other (Fox et al., 2013). Is the hippocampus important for dreaming as well as for mental time travel? A few years ago Llewellyn (2013) answered this question in the affirmative, mainly based on theoretical arguments linking REM sleep dreams with episodic memory. Many years previously an authoritative book of Solms (1997) had amassed considerable evidence in support of the notion that cessation or reduction of dreaming can occur after either a left posterior cortical lesion or a deep bilateral frontal lesion, but not after hippocampal lesions. In a commentary to Llewellyn’s article, Solms (2013) reiterated that it is a clinical fact that bilateral hippocampal lesions have no demonstrable effect on the occurrence of REM-like dreams. He wrote that he was looking forward to reading the vivid dream reports of the celebrated amnesic patient HM of Corkin, whose book had not yet appeared at the time. The book has now been out for some time and anyone can read in it that Henry Molaison’s dreams, if any, were by no means as vivid as expected by Solms. According to Corkin (2013), Henry’s dream reports were quite dull, merely describing images of houses and fields probably belonging to his old preoperatory memories, and such as to make Corkin suspect that they were merely anecdotes that he created on the spot in order to oblige his interlocutors. Corkin also makes the important point that Henry’s dream reports might have been warped by their 30 s span of immediate memory, after which the dream content was bound to evaporate. To our knowledge a thorough investigation of dreaming in amnesic patients with hippocampal lesions is still lacking, but a recent review presents several pieces of neuroimaging and electrophysiological evidence for an important participation of the hippocampus to dreaming process and to the contents of dreams (Cipolli et al., 2017). The hippocampus has probably a major role in providing the episodic memories, both recent and remote, that make up specific dream contents, while activation of the amygdala complex, anterior cingulate cortex and orbitofrontal cortex could instead be related to the emotional features of dreams.

## Epilog and Possible Developments

Consciousness is the expression of a global organization of the central nervous system which is subject to physiological modifications, as in dreamless sleep, to pharmacological alterations, as in general anesthesia, and to pathological disruptions, as in epilepsy, coma or vegetative state. These global states of neural organization or disorganization have been traditional objects of study of neurophysiology, neuropharmacology and clinical neurology. Neuropsychology has been more interested in specific aspects of higher brain function (perception, attention, memory, language, emotion, and so forth), and their disorders, rather than in consciousness *per se*. The evidence that the hippocampus may influence the temporal scope of thinking as well as the types of thought suggests that the neocortex may not be alone in the elaboration of conscious contents, and prompts further inquiries into the participation of subcortical structures to the multiple dimensions of consciousness, above and beyond a simple arousal function. For example, the cerebellum is generally considered to have little or no role in the neural organization underlying consciousness (e.g., Koch et al., 2016), in spite of the cognitive and affective deficits exhibited by patients with cerebellar lesions (Schmahmann, 2010). To our knowledge, studies similar to those of McCormick et al. (2018) on the influence of hippocampal lesions on conscious thinking have not been carried out on patients with cerebellar lesions. To be sure, the evidence for a role for the cerebellum in cognitive functions is rather weak compared to its major role in several forms of motor learning (Glickstein, 2007), and even large cerebellar lesions do not result in unconsciousness. However, also the ablation of an entire cerebral hemisphere appears to leave the patient with a conscious mind and a conscious sense of a personal self, regardless of which side is removed (Sperry, 1984). Though Sperry’s split-brain experiments are famous for suggesting a dimidiation of consciousness after section of the corpus callosum, Sperry himself has stated that attitudinal, orientational, emotional, contextual, semantic, and related cognitive components of conscious awareness stay unified in split-brain patients because they are mediated through undivided deep brain structures. The superior colliculus is almost certainly one of these structures (Corballis et al., 2018), but the contribution of other brainstem components remains to be investigated.

## Author Contributions

GB drafted the manuscript. CM discussed it and added changes and information.

## Conflict of Interest Statement

The authors declare that the research was conducted in the absence of any commercial or financial relationships that could be construed as a potential conflict of interest.
